# Serum Cardiotrophin-1 Concentration Is Negatively Associated with Controlled Attenuation Parameters in Subjects with Non-Alcoholic Fatty Liver Disease

**DOI:** 10.3390/jcm12072741

**Published:** 2023-04-06

**Authors:** Yi-Chun Liao, Juei-Seng Wu, Hsuan-Wen Chou, Hsin-Yu Kuo, Chun-Te Lee, Hung-Tsung Wu, Chung-Hao Li, Horng-Yih Ou

**Affiliations:** 1 Department of Internal Medicine, School of Medicine, College of Medicine, National Cheng Kung University, Tainan 701, Taiwan; 2 Division of Gastroenterology and Hepatology, Department of Internal Medicine, National Cheng Kung University Hospital, Tainan 703, Taiwan; 3 Division of Endocrinology and Metabolism, Department of Internal Medicine, National Cheng Kung University Hospital, Tainan 703, Taiwan; 4 Department of Family Medicine, An Nan Hospital, China Medical University, Tainan 709, Taiwan; 5 School of Medicine, College of Medicine, China Medical University, Taichung 404, Taiwan

**Keywords:** cardiotrophin-1, controlled attenuation parameter, diabetes, non-alcoholic fatty liver disease, obesity

## Abstract

Background: Since non-alcoholic fatty liver disease (NAFLD) is highly associated with obesity, cardiovascular disease, and diabetes, biomarkers for the diagnosis of NAFLD have become an important issue. Although cardiotrophin-1 (CT-1) has a protective effect on the liver in NAFLD animal models, the serum levels of CT-1 in human subjects with NAFLD were still unknown. Objective: The present study aimed to investigate the relationship between the circulating concentration of CT-1 and the severity of hepatic steatosis graded by the value of the controlled attenuation parameter (CAP) in humans. Design and Methods: The study was designed as a cross-sectional study, and a total of 182 subjects were enrolled. Hepatic steatosis measurement was carried out with a Firoscan^®^ device and recorded by CAP. The enrolled study subjects were categorized into CAP < 238 dB/m, 238 ≤ CAP ≤ 259 dB/m, 260 ≤ CAP ≤ 290 dB/m, and CAP > 290 dB/m. Serum CT-1 concentrations were determined by enzyme-linked immunosorbent assay. The association between the serum CT-1 concentration and NAFLD was examined by multivariate linear regression analysis. Results: Body mass index, percentage of body fat, systolic and diastolic blood pressure, alanine aminotransferase (ALT), cholesterol, triglyceride, hemoglobin A1c and homeostatic model assessment for insulin resistance (HOMA-IR) were significantly increased in groups with higher CAP value, whereas high-density lipoprotein cholesterol was significantly decreased. In addition, serum CT-1 concentrations were significantly decreased in subjects with higher CAP values. In multivariate linear regression models, including age, sex, body fat percentage, CAP, high sensitivity- C reactive protein, uric acid, creatinine, ALT, total cholesterol, and HOMA-IR, only age, CAP and uric acid independently associated with CT-1 levels. Moreover, having NAFLD was independently associated with CT-1 after adjustment for sex, obesity and type 2 diabetes. Conclusions: Serum CT-1 concentrations are decreased in subjects with NAFLD and negatively associated with CAP.

## 1. Introduction

Non-alcoholic fatty liver disease (NAFLD) is a spectrum of diseases ranging from hepatic steatosis, non-alcoholic steatohepatitis (NASH), hepatic fibrosis, cirrhosis to hepatocellular carcinoma (HCC), and it is currently highly prevalent all over the world, and responsible for 4% of all-cause mortality [1,2]. Liver disease-associated deaths are large because of the complications of cirrhosis and HCC, and the most common causes of cirrhosis worldwide are related to viral hepatitis, alcohol, and NAFLD. In addition, not only does NAFLD increase the risk of chronic liver diseases, but it is also highly associated with extrahepatic diseases, such as obesity, cardiovascular disease (CVD) and type 2 diabetes (T2DM) [3]. In view of the importance of NAFLD related to other metabolic disorders and the increasing prevalence of NAFLD, early diagnosis of NAFLD in the clinic becomes an important issue.

The diagnosis of NAFLD includes imaging or histological evidence of hepatic steatosis without the presence of significant alcohol consumption or other etiologies of liver diseases, such as viral hepatitis [4,5]. Liver biopsy is currently the gold standard for assessing and diagnosing the severity of hepatic steatosis. However, due to its high expense and invasiveness, various noninvasive measures, including ultrasound, and transient elastography, are used clinically for initial diagnosis [6,7,8,9]. Clinically, ultrasound is the first-line assessment for hepatic steatosis. However, it has inaccuracy in the exact quantification of fat accumulation and in reproducibility [10,11]. Computerized tomography is also limited in following up on NAFLD patients due to its attenuation value varying in different devices and its radiation exposure [10]. In addition, although magnetic resonance imaging (MRI) is still an accurate diagnostic device for the assessment of hepatic fat content [12,13], the high cost makes it less suitable for the point-of-care technique [8]. Controlled attenuation parameter (CAP) is one of the non-invasive methodologies based on signals acquired by Fibroscan^®^, which values are correlated well with the grading of hepatic steatosis [14,15,16,17]. Hepatic steatosis is graded on the basis of the percentage of fat within hepatocytes and divided into four groups: S0 (no steatosis, <5%), S1 (mild, 5~33%), S2 (moderate, 34~66%), and S3 (severe, >66%) [18]. 

Cardiotrophin-1 (CT-1), a member of the interleukin-6 family, was reported to have protective effects on the liver in experimental animals [19]. CT-1 induces liver regeneration through the promotion of angiogenesis and cell proliferation, offering protection from ischemia and reperfusion injury [19,20,21,22]. In addition, studies provided evidence that lower serum CT-1 concentration is associated with obesity [23], as well as impaired glucose utility and type 2 diabetes in humans [24]. Animal studies also showed that chronic administration of recombinant CT-1 improved hepatic steatosis in high-fat diet-induced NAFLD mice through inhibition of lipogenesis and enhancement of fatty acid breakdown [25].

In view of the increasing prevalence of NAFLD, discovering ideal biomarkers for the diagnosis of these diseases in prevention medicine has become an important issue in recent years. Although animal studies implied the beneficial effects of CT-1 on hepatic steatosis, the relationship between CT-1 and hepatic steatosis in humans remains unclear. Therefore, in this study, we investigated the association between the circulating concentration of CT-1 and liver steatosis grades determined by CAP in humans.

## 2. Materials and Methods

### 2.1. Study Subjects

The present study was designed as a cross-sectional study and performed after approval by the Institutional Review Board of the National Cheng Kung University Hospital. The subjects were not under any prolonged medical treatment, and none of the women were pregnant when tested were enrolled at the Health check-up center for general health examination in National Cheng Kung University Hospital since the study population from the Health checkup center might have fewer morbidities to affect the serum concentrations of CT-1. Exclusion criteria of the present study include (1) alcohol consumption ≥20 g per day for men and ≥10 g per day for women over the past year; (2) liver cirrhosis (liver stiffness measurement (LSM) > 12.5 kPa) or a history of hepatitis B or hepatitis C virus infection; (3) impaired nephritic function as determined by serum creatinine concentration over 1.5 mg/L; (4) any acute or chronic inflammatory diseases as determined by a leukocyte count over 10,000/mm^3^; (5) any other major diseases, including advanced malignant diseases.

The body weight, body height and waist circumstance (WC) were measured from patients in light clothing. The body height and weight of the study subjects were measured using a certified anthropometric instrument, and the body mass index (BMI; in kg/ m^2^) was calculated as weight (in kilograms) divided by height (in meters) squared for all subjects. BMI > 27 kg/m^2^ is defined as obesity according to the recommendations of the Health Promotion Administration in Taiwan. The body fat percentage was determined using bioelectrical impedance analysis (BIA) (InBody, Cerritos, CA, USA), which was based on the principle that the volume of a conductor is proportional to conductor length and inversely proportional to its electrical resistance by multi-frequency BIA method [26,27]. As for the blood pressure measurements, subjects were asked to rest in a sitting position with a supported back chair and measured twice at an interval of at least 5 min, with an appropriate-sized cuff wrapped around the upper arm using a DINAMAP vital sign monitor (Model 1846SX, Critikon, Irvine, CA, USA).

After 12 h of fasting, all participants received routine biochemical blood tests and the blood samples were collected in a vacutainer and sent to the laboratory of the Department of Pathology at National Cheng Kung University Hospital for the determination of blood aspartate aminotransferase (AST), alanine aminotransferase (ALT), creatinine, uric acid, cholesterol, triglyceride, high-density lipoprotein cholesterol (HDL-C) and hemoglobin A1c (HbA1c). The serum concentration of high-sensitivity C-reactive protein (hsCRP) (Immunology Consultants Laboratory, Newberg, OR, USA; with an intra-assay coefficient of variation (CV) of 2.9% and an inter-assay CV of 4.7%) and CT-1 (Abcam, Cambridge, UK; with an intra-assay CV of 5.7% and an inter-assay CV of 8.8%) were determined using commercial ELISA assay kits. The homeostatic model assessment of insulin resistance (HOMA-IR) was calculated by fasting plasma glucose (FPG) (mM) × fasting insulin (pg/mL)/22.5. Diabetes mellitus was defined by fasting plasma glucose of ≥7.0 mmol/L or 2 h post-load glucose of ≥11.1 mmol/L.

### 2.2. Liver Steatosis Measurement

Liver steatosis measurement was carried out with a Fibroscan^®^ device (Echosens, Créteil, France) by a certificated technician blinded to participants’ data and recorded by CAP. The examination was conducted using the standard M probe or XL probe, and the final CAP value was the median of the 10 individual measurements and was expressed in dB/m. Hepatic steatosis grade is decided by cutoff values of CAP, where CAP < 238 dB/m denotes no steatosis (S0), 238 ≤ CAP ≤ 259 dB/m denotes mild (S1), 260 ≤ CAP ≤ 290 dB/m denotes moderate (S2), and CAP > 290 dB/m denotes severe steatosis (S3), according to previous studies [28,29]. NAFLD was defined as the presence of hepatic steatosis on Fibroscan^®^ (CAP ≥ 238 dB/m) after the exclusion of secondary causes of hepatic steatosis (e.g., viral hepatitis, certain medications, and other medical conditions) and an alcohol consumption of ≥20 g/ day for men and ≥10 g/ day for women.

### 2.3. Statistical Analysis

Statistical product and service solutions (SPSS) software (ver 28.0; SPSS, Chicago, IL, USA) was used for all statistical analysis. All variables are expressed as the mean ± standard deviation (SD). Continuous variables were compared among groups using a one-way analysis of variance. Chi-square tests were used to compare categorical variables among groups. Differences in serum concentration of CT-1 between each steatosis grade were computed with the Bonferroni post hoc method and expressed with a scatter plot. A multiple linear regression analysis was conducted to identify variables that best-predicted serum CT-1 concentrations, of which the variable selection strategies were stepwise and backward. A *p* value < 0.05 was considered statistically significant.

## 3. Results

**Figure 1:**
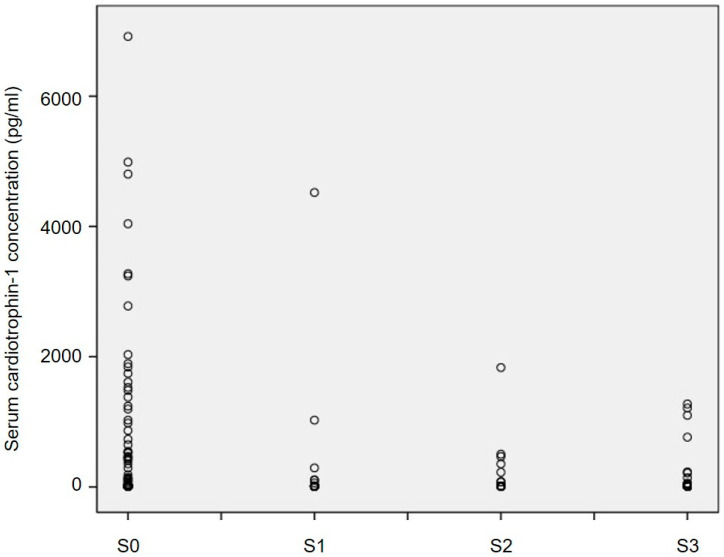
Serum concentrations of cardiotrophin-1 (CT-1) in subjects with NAFLD. Box and whisker plot of serum CT-1 concentrations in subjects with CAP < 238 dB/m (S0; *n* = 90), 238 ≤ CAP ≤ 259 dB/m (S1; *n* = 27), 260 ≤ CAP ≤ 290 dB/m (S2; *n* = 23), and CAP > 290 dB/m (S3; *n* = 42). The line inside the box represents the median of the distribution, the box top and bottom values are defined by the 25th and 75th percentiles, and the whiskers are minimum and maximum values.

In this study, a total of 182 subjects were enrolled and categorized into four steatosis grades based on CAP values, where 90 subjects were in the S0 group (CAP < 238 dB/m), 27 subjects were in S1 (238 ≤ CAP ≤ 259 dB/m), 23 subjects were in S2 (260 ≤ CAP ≤ 290 dB/m), and 42 subjects were in S3 group (CAP > 290 dB/m). The baseline characteristics of subjects are shown in Table 1. Statistical differences were not observed in age, sex, body height, hsCRP, and creatinine among groups. We found that the body weight, BMI, WC, body fat percentage, systolic blood pressure, diastolic blood pressure, AST, ALT, AST/ALT ratio, uric acid, cholesterol, triglyceride, HbA1C, and HOMA-IR were significantly elevated as steatosis grades worsen from no steatosis to severe steatosis, and CT-1 (S0: 624.67 ± 1245.38, S1: 235.40 ± 879.51, S2: 162.17 ± 394.31, S3: 129.74 ± 324.28; *p* = 0.018 for trend) and HDL-C was significantly decreased. The serum concentration of CT-1 was significantly decreased in S3, as compared with the S0 group (*p* = 0.04), while no differences were observed between any other two groups (Figure 1).

As shown in Figure 1, the values of serum CT-1 concentrations were significantly decreased with increased CAP values without adjusting for obesity and diabetes. Since previous studies indicated that both obesity and diabetes are confounding factors that affect the levels of CT-1, the relationship between CT-1 and CAP was therefore investigated after adjustment of the confounding factors, as shown in Table 2. To further explore independent factors associated with serum cardiotrophin-1 concentrations, we performed a multivariate linear regression analysis (Table 2). Firstly, in Model 1 of Table 2, we investigated the relationship between serum CT-1 concentration and CAP. After adjusting sex and body fat, we found age (β = −25.56, 95% CI = −38.93~−12.18, *p* < 0.001) and CAP (β = −3.75, 95% CI = −6.80~−0.70, *p* = 0.016) had significant associations with CT-1 value (Model 1). Since previous studies indicated that both obesity and diabetes are confounding factors that affect the levels of CT-1, the relationship between CT-1 and CAP was therefore investigated after further adjustment for the confounding factors as shown in Model 2 of Table 2, having NAFLD (β = −374.76, 95% CI = −670.88~−78.65, *p* = 0.013) was independently associated with CT-1 after adjustment for sex, obesity, and diabetes (Model 2). In order to confirm the relationship between serum CT-1 and CAP, we further adjusted additional confounders, including sex, body fat, hsCRP, uric acid, creatinine, ALT, cholesterol, and HOMA-IR, as shown in Model 3, and still found that the age (β = −22.46, 95% CI = −35.98~−8.94, *p* = 0.001) and CAP (β = −3.08, 95% CI = −6.16~−0.011, *p* = 0.049) were independently associated with CT-1 value (*p* = 0.049). After the adjustment for these additional confounders, we thus confirmed the negative relationship between serum CT-1 concentration and CAP in conclusion.

## 4. Discussion

To the best of our knowledge, this is the first study investigating correlations between CT-1 and hepatic steatosis in humans. In our study, we found that serum CT-1 concentrations were significantly decreased in subjects with hepatic steatosis, and CAP values are negatively and independently associated with serum CT-1 concentrations.

Apart from all the imaging techniques, serum biomarkers can also be used as a novel strategy for the diagnosis of NAFLD. A number of hepatokines, including fibrinogen-like protein-1 (FGL-1, also called hepassocin or hepatocyte-derived fibrinogen-related protein 1) [30], and fetuin-A [31] were investigated to play crucial pathophysiological roles in the development of NAFLD. In addition, the serum concentrations of these hepatokines were reported to be changed in human subjects with NAFLD. Moreover, these hepatokines also play important roles in the link between obesity [32], type 2 diabetes [33] and NAFLD. In addition to FGL-1 and fetuin-A, numerous studies have demonstrated that CT-1 plays a role in NAFLD, and it exerts beneficial effects on organ protection, including the liver. Administration of CT-1 recombinant protein in mice inhibited de novo lipogenesis and stimulated fatty acid oxidation in hepatocytes through activation of adenosine 5′-monophosphate (AMP)-activated protein kinase [25,34]. CT-1 administrations also promoted angiogenesis and cell proliferation to improve the hepatic function in cirrhosis [21]. In addition, CT-1 repressed inflammation and reduced ischemia injury during liver transplants [22,35]. In the present study, we found that serum CT-1 levels decreased significantly in patients with hepatic steatosis compared to those without steatosis and are negatively related to CAP values. Our results indicated that CT-1 has the potential to act as a biomarker for clinical diagnosis. 

Although the present study is the first to evaluate the serum concentrations of CT-1 in human subjects with NAFLD, the levels of CT-1 had been determined in human subjects with obesity, impaired arterial stiffness, and diabetes. It was found that circulating CT-1 levels were significantly higher in subjects with brachial-ankle pulse wave velocity (baPWV) > 1400 than those with baPWV ≤ 1400 cm/s and it is positively related to baPWV independent of traditional cardiometabolic risk factors for arterial stiffness [36]. In addition, CT-1 is increased in subjects with impaired glucose tolerance and newly diagnosed diabetes, and impaired glucose tolerance and newly diagnosed diabetes were positively associated with CT-1 concentrations [24]. Intriguingly, non-diabetic subjects who were overweight or obese had significantly lower CT-1 concentrations than those with normal weight, and both obesity and being overweight were inversely associated with CT-1 levels [23]. On the other hand, it was reported that CT-1 expressions were increased in the liver of patients with NAFLD and in steatotic livers from genetic and diet-induced diabetic animal models [25]; however, without considering the confounding factors, such as obesity and diabetes, the levels of CT-1 in NAFLD were still obscure. In the present study, we found that NAFLD is a critical, independent associated factor that correlates with CT-1 after adjusting for obesity and diabetes.

CT-1 promotes cell survival and proliferation to provide myocardial protection, and it predisposes the heart to pathological conditions. Although CT-1 is predominantly expressed and synthesized in the heart, it has been detected in several tissues, such as the liver, adipose tissue, and tissues in the respiratory and nervous systems [37]. Thus, multiple tissues that expressed and secreted CT-1 might affect the serum concentrations of CT-1 in NAFLD. On the other hand, a previous study enrolled 20 study subjects to investigate the levels of CT-1 in the liver of subjects with NAFLD, and they found that the hepatic CT-1 mRNA expressions were increased in subjects with NAFLD without adjustment for any possible confounding factors [25]. In contrast to the observation in a previous study, we increased the sample size and surveyed the serum concentrations of CT-1 in subjects with NAFLD, and we found that the serum concentrations of CT-1 were negatively associated with CAP value after adjustment for confounding factors. In addition, different pathologies, such as the genetics of hepatic lipid metabolism, hepatic de novo lipogenesis and dysfunctional adipose tissue, can result in the same phenotype, hepatic steatosis [38]. Although treatment of recombinant CT-1 to ob/ob diabetic mice resulted in upregulation in hepatic genes promoting β-oxidation and downregulation in lipogenic gene expressions, it was found that CT-1 has an activity to stimulate lipolysis through the regulation of lipases [39]. In addition, previous studies indicated the anti-steatotic role of CT-1 in NAFLD driven by genetic components and dysfunctional adipose tissue. A downregulation of adipokines, including resistin and leptin [40,41], involved in the progression of hepatic steatosis was observed after the administration of CT-1 recombinant protein [42]. A decreased level of CT-1 in patients with hepatic steatosis might be related to the progression of NAFLD driven by genetic components and dysfunctional adipose tissue. Moreover, CT-1 levels might be related to insulin resistance in subjects with metabolic syndrome [43]; thus, we speculated that the timing of progression of steatosis to metabolic syndrome or type 2 diabetes mellitus with insulin resistance status was relevant to the CT-1 levels. Although previous studies suggested that CT-1 is increased in impaired glucose tolerance, diabetes, and those who had higher baPWV, our study subjects were free from the above conditions. They were mostly of normoglycemia (with a mean HbA1C of 5.4~5.8%) and younger in age (mean 43~47 years) and thus presumed to have relatively normal baPWV, although we did not measure it. Furthermore, the negative association between CT-1 and NAFLD in our study is compatible with one previous study showing that in non-diabetic subjects, obesity and being overweight were inversely associated with CT-1 levels [23]. In addition, the crosstalk of adipose tissue and liver in the development of hepatic steatosis by CT-1 still needs further studies to investigate in detail. 

There are several limitations in the present study. First, this is a cross-sectional study, so causal relationships could not be established, and the sample size is relatively small; we conducted a detailed statistical analysis by adjusting multiple potential confounding factors to overcome. Second, CT-1 is not only recognized as an adipokine but also serves as a hepatokine [44]. Since it was known that obesity is negatively associated with CT-1 levels, we therefore, adjusted obesity and the percentage of body fat content and found that NAFLD was still an independent factor with CT-1 levels. On the other hand, it was known that increased visceral fat content plays a key role in the development of obesity, NAFLD, and metabolic syndrome [45]. However, since magnetic resonance imaging was not used in the present study to precisely measure body fat mass and fat distribution, further studies are needed to investigate the role of body fat mass and fat distribution in the relationship between CT-1 levels and NAFLD. Third, we used Fibroscan to diagnose NAFLD in our study subjects. However, NASH can be only diagnosed by liver biopsy-proven steatosis, inflammatory infiltrates, and ballooning degeneration of hepatocytes. Thus, the NAFLD subjects with NASH were not excluded and also probably be included in our study. Furthermore, we excluded subjects with liver cirrhosis (but not fibrosis) is that the decompensated state would possibly interfere with measures of several interested parameters. Finally, since all of the subjects are Han Chinese, the result may not be generalizable to other populations. 

## 5. Conclusions

Serum CT-1 concentration is significantly decreased in subjects with NAFLD and is negatively associated with CAP and NAFLD. Future studies are needed to validate whether CT-1 levels could be used as a biomarker of NAFLD.

## Figures and Tables

**Table 1:** Clinical characteristics of the enrolled study subjects.

Variables	CAP < 238	238 ≤ CAP ≤ 260	260 ≤ CAP ≤ 290	CAP > 290	*p*
*n*	90	27	23	42	
Age (years)	43.3 ± 9.8	44.15 ± 11.66	47.78 ± 9.27	47.3 ± 10.63	0.094
Sex (male, %)	48	63	61	67	NS
Body height (cm)	164.94 ± 8.18	165.92 ± 8.69	165.57 ± 9.7	166.46 ± 8.28	NS
Body weight (kg)	61.27 ± 11.05	66.99 ± 11.32	70.11 ± 16.97	75.1 ± 13.3	<0.001
BMI (kg/m^2^)	22.45 ± 3.25	24.18 ± 2.36	25.25 ± 3.77	27.01 ± 3.89	<0.001
WC (cm)	74.49 ± 8.6	76.69 ± 6.71	82.74 ± 10.88	87.65 ± 9.7	<0.001
Body fat (%)	23.52 ± 5.75	25.38 ± 5.77	25.41 ± 5.82	28.23 ± 5.9	<0.001
SBP (mmHg)	113.72 ± 14.46	120.11 ± 10.54	119.26 ± 16.43	124.75 ± 12.28	<0.001
DBP (mmHg)	67.49 ± 10.34	71.98 ± 8.19	71.33 ± 10.89	75.6 ± 8.67	<0.001
hsCRP (mg/L)	1.49 ± 2.28	2.16 ± 2.51	2.22 ± 2.41	2.93 ± 3.92	0.074
AST (U/L)	20.62 ± 9.64	25.22 ± 10.07	27.74 ± 11.88	48.21 ± 35.46	<0.001
ALT (U/L)	21.16 ± 6.23	21.44 ± 4.76	22.61 ± 5.6	31.6 ± 14.82	<0.001
AST/ ALT	0.96 ± 0.29	1.15 ± 0.29	1.21 ± 0.37	1.42 ± 0.5	<0.001
Creatinine (mg/dL)	0.76 ± 0.18	0.70 ± 0.21	0.73 ± 0.22	0.72 ± 0.19	NS
Uric acid (mg/dL)	5.68 ± 1.54	6.02 ± 1.41	6.44 ± 1.97	6.9 ± 1.33	<0.001
Cholesterol (mg/dL)	187.01 ± 34.36	195.44 ± 39.11	204.52 ± 36.68	203.41 ± 36.98	0.044
Triglyceride (mg/dL)	96.19 ± 48.93	106.92 ± 55.26	129.04 ± 69.79	176.67 ± 114.3	<0.001
HDL-c (mg/dL)	56.19 ± 14.39	53.0 ± 9.67	51.30 ± 14.51	46.92 ± 12.74	0.004
HbA1C (%)	5.43 ± 0.27	5.37 ± 0.26	5.88 ± 1.29	5.76 ± 0.63	<0.001
HOMA-IR	0.02 ± 0.20	0.03 ± 0.03	0.06 ± 0.05	0.08 ± 0.14	<0.001

NS, no significance; CAP, controlled attenuation parameter; BMI, body mass index; WC, waist circumference; SBP, systolic blood pressure; DBP, diastolic blood pressure; hsCRP, high-sensitivity C-reactive protein; AST, aspartate aminotransferase; ALT, alanine aminotransferase; HDL-C, high-density lipoprotein cholesterol; HbA1C, hemoglobin A1c; HOMA-IR, homeostatic model assessment of insulin resistance.

**Table 2:** Results of multivariate linear regression analysis between CT-1 and clinical variables.

Variables	Model 1 β (95% CI)	*p*-Value	Model 2 β (95% CI)	*p*-Value	Model 3 β (95% CI)	*p*-Value
age	−25.56 (−38.93, −12.18)	<0.001	−25.56 (−39.16, −11.97)	<0.001	−22.46 (−35.98, −8.94)	0.001
sex	188.42 (−150.63, 527.48)	0.274	183.34 (−96.73, 463.43)	0.198	335.09 (−133.04, 803.23)	0.159
body fat percentage	2.65 (−26.59, 31.89)	0.858			−3.95 (−37.21, 29.30)	0.815
CAP	−3.75 (−6.80, −0.70)	0.016			−3.08 (−6.16, −0.011)	0.049
Obesity (yes vs. no)			−105.92 (−475.01, 263.17)	0.572		
NAFLD (yes vs. no)			−374.76 (−670.88, −78.65)	0.013		
diabetes (yes vs. no)			−68.43 (−794.55, 657.68)	0.853		
hsCRP					40.78 (−11.38, 92.94)	0.125
uric acid					86.57 (−36.97, 210.12)	0.0168
creatinine					−901.51 (−2000.41, 197.38)	0.107
ALT					−12.14 (−27.17, 2.88)	0.112
cholesterol					−1.71 (−5.71, 2.29)	0.400
HOMA-IR					−765.22 (−4841.56, 3311.12)	0.711

Dependent variable: serum CT-1 concentration; CAP, controlled attenuation parameter; NAFLD, non-alcoholic fatty liver disease; hsCRP, high sensitivity C-reactive protein; ALT, alanine aminotransferase; HOMA-IR, homeostatic model assessment of insulin resistance.

## Data Availability

The data that support the findings of this study are available from the corresponding author upon reasonable request.
